# Exploring the top 30 drugs associated with drug-induced thrombotic microangiopathy based on the FDA adverse event reporting system

**DOI:** 10.3389/fphar.2025.1658963

**Published:** 2025-09-19

**Authors:** Yi Yin, Fanmin Meng, Yanjiao Fan

**Affiliations:** ^1^ Department of Pediatric Intensive Care Unit, Shandong Provincial Hospital Affiliated to Shandong First Medical University, Jinan, Shandong, China; ^2^ Department of Clinical Laboratory, Zibo First Hospital, Zibo, Shandong, China; ^3^ Department of Pediatrics, Zibo First Hospital, Zibo, Shandong, China

**Keywords:** FDA adverse event reporting system (FAERS), thrombotic microangiopathy, TMA, pharmacovigilance, adverse event (AE)

## Abstract

**Background:**

Drug-induced thrombotic microangiopathy (TMA) significantly impacts patient health and quality of life. This study aims to conduct an exploratory analysis of TMA reports and the most frequently associated drugs in the FDA Adverse Event Reporting System (FAERS) database.

**Methods:**

We analyzed FAERS reports associated with TMA from the first quarter of 2004 to the fourth quarter of 2024. A disproportionality analysis was conducted to detect significant safety signals. Potential causative drugs were identified, and the top 30 medications with the highest number of TMA reports and strongest signal strengths were ranked accordingly.

**Results:**

Analysis of 22,375,298 reports in the FAERS database identified 13,748 cases of thrombotic microangiopathy (TMA). Among the top 30 medications potentially associated with drug-induced TMA, antineoplastic and immunomodulatory agents predominated both in reporting frequency and signal strength metrics. Disproportionality analysis specifically revealed multiple drugs not currently labeled for TMA risk, with antineoplastic agents comprising the majority. Notably, several less frequently implicated agents - including micafungin, foscarnet, ketoprofen, and atovaquone - also demonstrated significant associations. These pharmacovigilance signals require cautious interpretation given the inherent limitations in establishing definitive causality through spontaneous reporting data.

**Conclusion:**

Our comprehensive analysis of drug rankings and signal strengths associated with TMA in FAERS underscores the critical role of pharmacovigilance in identifying and understanding drug-induced TMA. These findings necessitate further research to validate the observed associations and to develop effective risk management strategies, ultimately improving patient outcomes. This study provides valuable evidence to support the accurate clinical identification of drug-related TMA.

## 1 Introduction

Thrombotic microangiopathy (TMA) is a pathological syndrome characterized by hemolytic anemia, thrombocytopenia, and microvascular thrombosis (George and Nester, 2014). Multiple etiologies of TMA have been described including autoantibodies against ADAMTS13 (causing thrombotic thrombocytopenic purpura), infections [most commonly with Shiga toxin producing *E. coli* O157 producing hemolytic uremia syndrome (HUS)], mutations of the complement regulatory pathways (producing atypical HUS), and drug-induced TMA (George and Nester, 2014; Reese et al., 2015). A previous study indicated that drug-induced thrombotic microangiopathy represented 10%–15% of all TMA cases and was probably under-reported (Mazzierli et al., 2022).

While there are specific treatments for other types of TMA, such as eculizumab for atypical HUS (aHUS), and caplacizumab and plasma exchange for thrombotic thrombocytopenic purpura (TTP), supportive care and avoidance of trigger drugs are the only known beneficial management strategies for drug-induced TMA (Reese et al., 2015). Therefore, identification and withdrawal of the causative drug is key.

The current research on drug-induced TMA is mainly focused on case reports and systematic reviews (Al-Nouri et al., 2015; Chen et al., 2016; Saleem et al., 2018, pp. 2014–2018; Khan et al., 2023, pp. 2018–2023). The latest information on drugs potentially associated with TMA in the real world remains limited. A previous study had utilized the Japanese Spontaneous Reporting System database to analyze drugs potentially associated with TMA (Niinomi et al., 2020). However, to the best of our knowledge, no studies have employed the FDA adverse Event Reporting System (FAERS) database to identify drugs potentially associated with TMA, FAERS is the largest adverse event reporting database (Rodriguez et al., 2001), and has been widely recognized in post-marketing drug surveillance (Dhodapkar et al., 2022; Salah et al., 2024). Our research aims to address this gap by identifying medications linked to an increased risk of TMA. This analysis can uncover abnormal reporting frequencies and offer valuable insights into potential safety signals, thereby informing clinical practice and regulatory decision-making.

## 2 Materials and methods

### 2.1 Data source and collection

Data for this study were obtained from the FAERS database from the first quarter of 2004 to the fourth quarter of 2024. This is the U.S. Food and Drug Administration’s post-marketing safety monitoring program for all marketed drugs and therapeutic biologics that contains adverse event (AE) reports submitted by healthcare professionals, consumers, and manufacturers, providing a comprehensive overview of real-world AE occurrence. Currently, the FAERS database publicly posts all adverse event reports received by the Food and Drug Administration (FDA) since 2004 and is updated quarterly.

The FAERS data files consist of seven databases, namely, demographic and administrative information (DEMO), adverse drug reaction information (REAC), patient outcome information (OUTC), drug information (DRUG), drug therapy start and end dates (THER), information on report sources (RPSR), and indications for use/diagnosis (INDI). According to FDA guidelines, we removed duplicate reports. If cases had the same case ID, the latest report with FDA_DT was retained, and if the case ID and FDA_DT were the same, the report with the higher PRIMARYID was retained. We obtained all PTs for TMA in MedDRA (version 27.1) and used them in subsequent analyses to ensure that the PTs analyzed were authentic from a clinical perspective. The detailed PTs including thrombotic microangiopathy (10,043,645), renal-limited thrombotic microangiopathy (10,085,346), pulmonary tumor thrombotic microangiopathy (10,079,988), thrombotic thrombocytopenic purpura (10,043,648), hemolytic uremic syndrome (10,018,932), atypical haemolytic uraemic syndrome (10,079,840).

### 2.2 Data analysis

Disproportionality analysis was considered a pivotal tool for evaluating potential associations between specific AEs and particular drugs (Dores et al., 2021). In this study, the reporting odds ratio (ROR), the proportional reporting ratio (PRR), the Bayesian confidence propagation neural network (BCPNN), and the empirical Bayesian geometric mean (EBGM) were used to calculate the signal strength. The results of adverse reaction signals should meet the positive signal discrimination criteria of the above four algorithms (Supplementary Table S1), leveraging their respective strengths to enhance the detection of potential rare adverse reactions and validate results from different perspectives. Descriptive analysis was used to summarize and present the clinical characteristics of the patients in drug-induced TMA reports. All statistical analyses were conducted using R_4.4.1 software.

## 3 Results

### 3.1 Descriptive analysis

**FIGURE 1:**
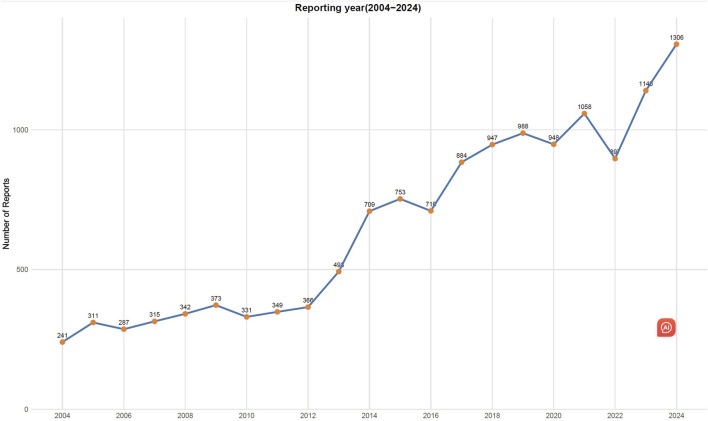
Number of reported cases of drug-induced thrombotic microangiopathy from Q1 2004 to Q4 2024.

From 1 January 2004, to 31 December 2024, a total of 22,375,298 AEs were reported in the FAERS database, of which 13,748 were related to TMA. The clinical characteristics of drug-related TMA are shown in Table 1. In terms of sex, there were 6232 female cases (45.3%), 5483 male cases (39.9%), and 2033 cases (14.8%) with partial sex information missing. The patients had a mean age of 47.5 years and a mean weight of 65.4 kg. In addition, the majority of reports (81.8%)were from healthcare professionals. The United States, Japan and France reported the highest number of TMA cases (4,631, 1,831 and 1,592 reports, respectively). The overall trend of drug-related TMA reports from 2004 to 2024 shows an increase with notable fluctuations (Figure 1).

**TABLE 1:** Basic information of adverse reaction reports related to TMA in the FAERS database.

Variables	Reports, n (%)
Sex	
Female	6232 (45.3%)
Male	5483 (39.9%)
Unknown	2033 (14.8%)
Age (years)	
Mean (SD)	47.5 (21.3)
Unknown	3165 (23.0%)
Weight (kg)	
Mean (SD)	65.4 (25.4)
Unknown	11072 (80.5%)
Occupation of the reporter	
Physician	5189 (37.7%)
Other health-professional	2974 (21.6%)
Health professional	2542 (18.5%)
Pharmacist	554 (4.0%)
Consumer	1752 (12.7%)
Unknown	686 (5.0%)
Lawyer	51 (0.4%)
Reported Countries (top 5)	
United States	4631 (33.7%)
Japan	1831 (13.3%)
France	1592 (11.6%)
Germany	636 (4.6%)
Canada	601 (4.4%)
United Kingdom	575 (4.2%)

Abbreviations: TMA, thrombotic microangiopathy; FAERS, FDA, adverse event reporting system.

### 3.2 Disproportionality analysis

**FIGURE 2:**
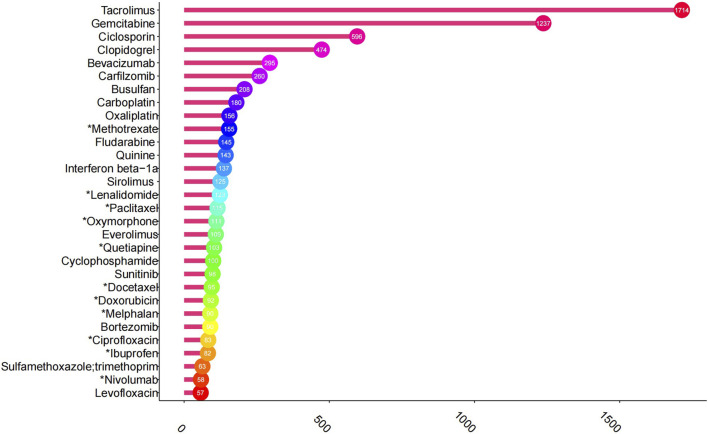
Top 30 drugs with the highest number of reported thrombotic microangiopathy. *The package insert did not suggest risk for thrombotic microangiopathy.

Based on the frequency of AE reports, the top 30 drugs potentially associated with TMA are detailed in Figure 2. Tacrolimus is the most commonly reported medication with 1,714 cases, followed by gemcitabine (1237 cases), ciclosporin (596 cases), clopidogrel (474 cases), bevacizumab (295 cases), carfilzomib (260 cases), busulfan (208 cases), carboplatin 180 cases), oxaliplatin (156 cases), and methotrexate (155 cases).

**FIGURE 3:**
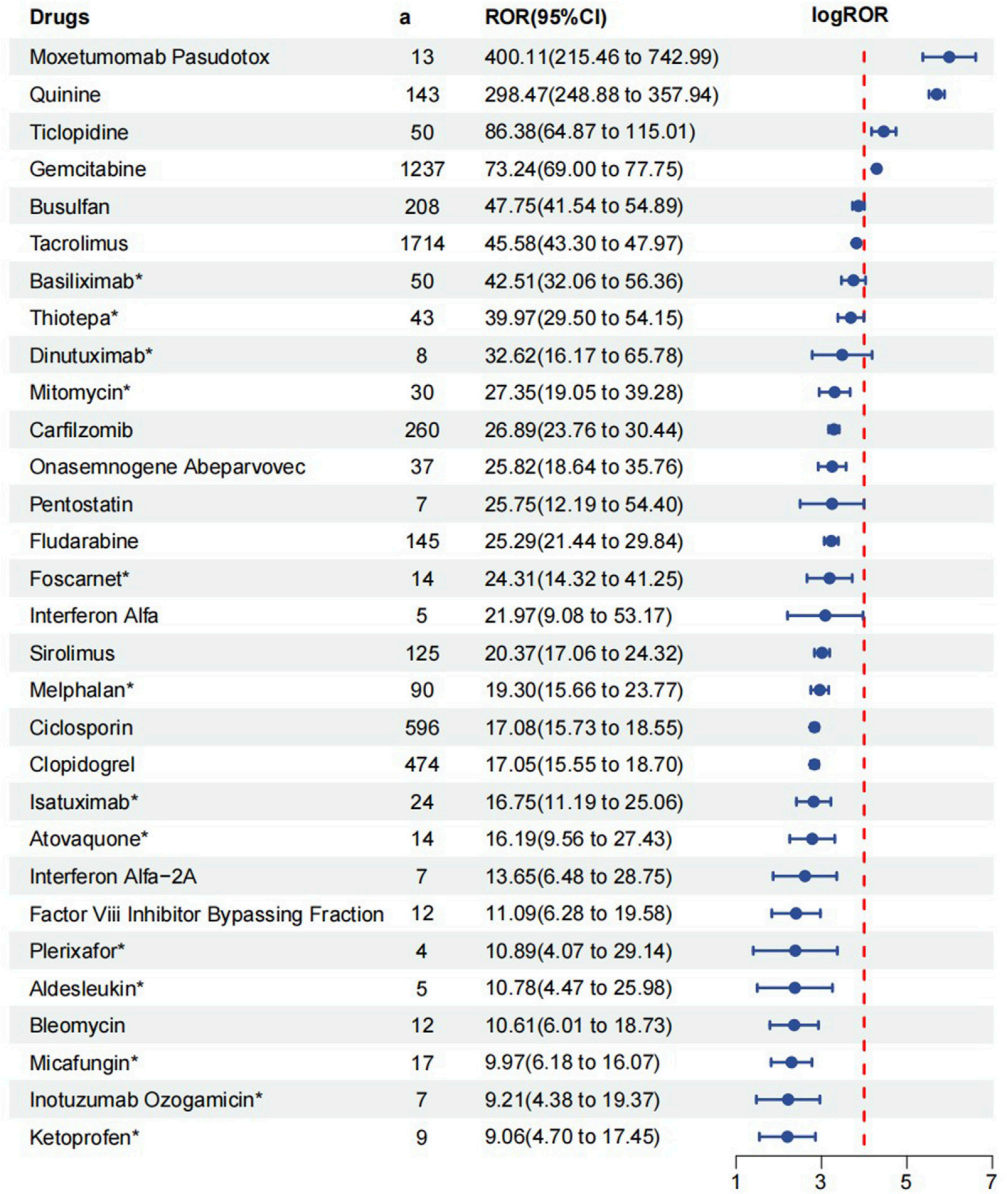
Top 30 drugs for signal strength. *The package insert did not suggest risk for thrombotic microangiopathy.

The top 30 drugs are listed in Figure 3 according to ROR signal strength based on the disproportionality analysis of four algorithms. Notably, 17 of these drugs list TMA risk in their package inserts, while the remaining 13 do not. These 13 drugs are basiliximab, thiotepa, dinutuximab, mitomycin, foscarnet, melphalan, isatuximab, atovaquone, plerixafor, aldesleukin, micafungin, inotuzumab ozogamicin and ketoprofen.

**FIGURE 4:**
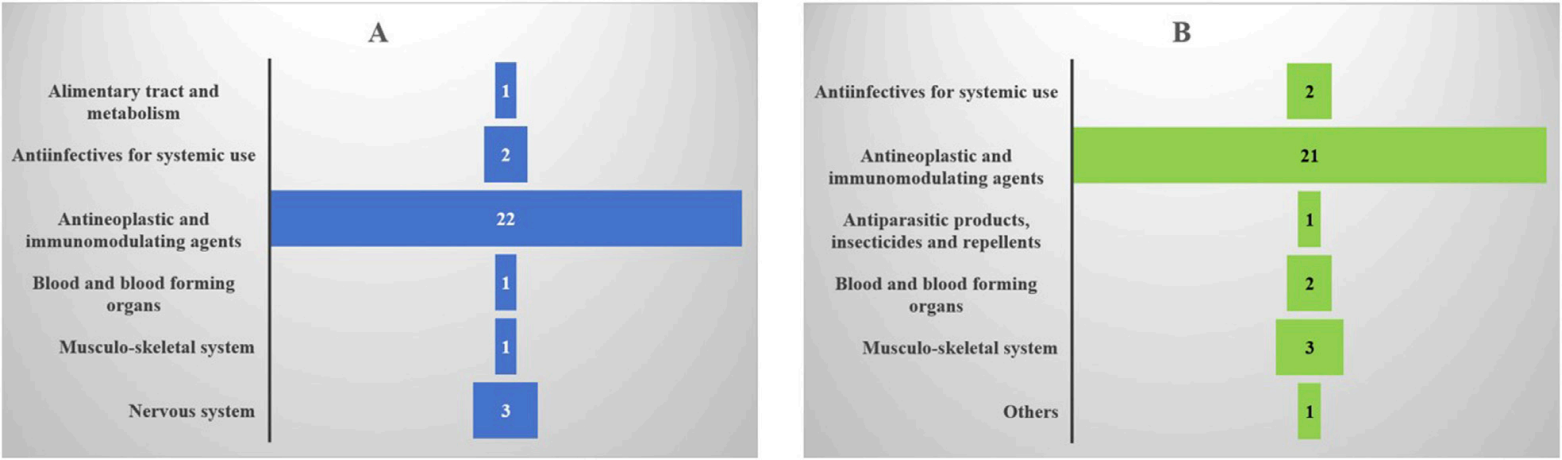
Top 30 drug-induced thrombotic microangiopathy classified by Anatomical Therapeutic Chemical (ATC) system. **(A)** Reporting cases; **(B)** Signal strength).

We categorized the drugs by the Anatomical Therapeutic Chemical (ATC) classification system (Figure 4) and found that, both in terms of case numbers and signal strength, antineoplastic and immunomodulating agents were the most commonly associated with TMA.

## 4 Discussion

Thrombotic microangiopathy (TMA) was first described in 1924. It can be hereditary or acquired, with onset being either sudden or insidious. If not promptly diagnosed and treated, it may lead to severe complications such as hypertension and renal failure, which can be life-threatening (George and Nester, 2014). Among its subtypes, drug-induced TMA is particularly significant due to its complex underlying mechanisms, which may be triggered via pharmacological pathways such as immune-mediated reactions or toxic dose-related effects (George and Nester, 2014).

Our study observed a year-by-year increase in the reported cases of TMA, suggesting improved public awareness of this condition. Interestingly, the majority of adverse reaction reports (81.8%) were submitted by healthcare professionals, which confirms the reliability of this study to some extent. The occurrence of TMA can severely impact patient health, underscoring the need for heightened vigilance among healthcare professionals.

In our study, we observed a significantly higher number of drug-induced TMA cases reported in females compared to males. One potential explanation for this disparity is that thrombotic thrombocytopenic purpura (TTP) is ∼2 fold more frequent in women and its outcome is characterized by a relapsing tendency (Khan et al., 2023). Additionally, most reports originated from the United States (33.7%), suggesting potential regional or cultural differences in reporting practices that may obscure true associations and warrant further investigation.

Our study comprehensively evaluated the real-world AE reports of drug-related TMA using the FAERS database. We detailed the clinical characteristics of these cases and identified the drugs most strongly associated with TMA.

In terms of the frequency of AE reports, tacrolimus, gemcitabine, and cyclosporine were the three most commonly implicated drugs, which aligns with previous case reports and meta-analyses (Al-Nouri et al., 2015; Mazzierli et al., 2022). Methotrexate-associated TMA has only been reported in the literature (Pliquett et al., 2020), whereas its prescribing information mentions thrombocytopenia but omits TMA. High-dose methotrexate-induced nephrotoxicity (Widemann et al., 2004; Wiczer et al., 2016), combined with its inherent potential to cause hemolysis (Sattar et al., 2022), may increase the risk of hemolytic uremic syndrome (HUS). This mechanism could partially explain methotrexate-associated TMA.

Based on imbalance analysis with four algorithms, we identified the top 30 medications with significant signals and compared them against their labelling information. To some extent, the more robust the observed adverse drug reaction signal, the greater the clinical concern should be regarding its potential to induce TMA (Li et al., 2024). Antineoplastic/immunomodulatory agents showed the highest disproportionality signals for TMA, including carfilzomib, busulfan, fludarabine, and sirolimus—all of which are documented in their product labels (Giralt et al., 2003; Shimoni et al., 2004; Sakashita et al., 2024).

Additionally, clopidogrel (ATC classification: Blood and blood forming organs) and quinine (ATC classification: Musculo-skeletal system) also demonstrated significant signal strength for TMA (Pisoni et al., 2001; Al-Nouri et al., 2015; Tada et al., 2016; Page et al., 2017), reinforcing the validity of our study.

Due to small sample sizes and short durations, pre-marketing trials often struggle to detect delayed or rare adverse reactions. However, these limitations can be mitigated by analyzing the FAERS database. Consequently, we focused particularly on the 13 drugs whose prescribing information did not mention TMA as an adverse reaction.

Among these 13 drugs, antineoplastic and immunomodulating agents comprised nine of them. The relationship between TMA and antineoplastic therapy was first reported in 1972 by Liu et al. after the application of mitomycin in a patient with metastatic tumors (Liu et al., 1971). Surprisingly, over the past few decades, the incidence of cancer drug-induced thrombotic microangiopathy (TMA) has reached approximately 15% (Izzedine and Perazella, 2015), significantly higher than that observed in the general population for atypical HUS (0.5–2 in 1,000,000) (Constantinescu et al., 2004; Loirat and Frémeaux-Bacchi, 2011) and TTP (3.8 in 1,000,000) (Miller et al., 2004). In clinical practice, hematologic abnormalities in cancer patients are often attributed to myelosuppression, which may lead to delayed diagnosis. Therefore, it is particularly crucial to enhance awareness of TMA events associated with antineoplastic agents.

Micafungin is an echinocandin antifungal agent that exerts its effect by inhibiting β-(1,3)-D-glucan synthase, thereby disrupting fungal cell wall synthesis (Denning, 2003). The pathogenesis of micafungin-induced TMA may be similar to that of ticlopidine-induced autoantibodies, where ticlopidine leads to loss of ADAMTS13 function or induces other mechanisms that alter ADAMTS13 activity (Nazzal et al., 2011). Although TMA is not explicitly listed as an adverse reaction in micafungin’s prescribing information, this antifungal agent has been reported to cause hemolysis as well as renal impairment or acute renal failure (FDA-Approved Drugs, 2025). Therefore, the prescribing information for this drug specifies that patients who develop hemolysis or hemolytic anemia during micafungin therapy should undergo close monitoring of relevant laboratory parameters and clinical manifestations.

Foscarnet is an antiviral medication primarily used to treat severe viral infections. An 8-year-old girl with myelodysplastic syndrome (refractory cytopenia) developed TMA after receiving foscarnet treatment on day 40 post unrelated donor bone marrow transplantation (Miyamoto et al., 2018).

Reports of TMA associated with ketoprofen and atovaquone are currently limited. The identification of these new associations may be attributed to the comprehensive nature of our study, which analyzed a large, real-world dataset from the FAERS database. The FAERS database contains spontaneous reports of adverse events submitted by healthcare professionals, patients, and pharmaceutical companies, allowing for the detection of rare or previously unknown drug-related adverse events. Although this type of analysis cannot determine AE incidence or establish causality, these findings elucidate the AEs reported in patients treated with ketoprofen or atovaquone, suggesting drug-AE associations warrant further investigation.

Our study has several limitations. Firstly, considering that FAERS is a spontaneous reporting system, issues such as underreporting, misreporting, and incomplete reporting are inevitable, which may introduce bias into the conclusions. Secondly, the significant signals we identified cannot confirm a direct causal relationship between the drugs and TMA. As per established diagnostic criteria (Mazzierli et al., 2022), to confirm a drug as the causative agent of TMA, a close temporal relationship between drug exposure and TMA onset must be established, and other potential causes of TMA (e.g., viral infections, malignancies, autoimmune diseases, sepsis, hypertension or concomitant drugs) must be rigorously excluded (Izzedine and Perazella, 2015). In many cases within the database, these criteria cannot be verified due to incomplete information. Furthermore, definitive confirmation often requires supporting evidence from preclinical models or histopathological data. Therefore, our findings should be interpreted as generating hypotheses about potential risk, which require further validation through well-designed pharmacoepidemiological studies and careful clinical assessment of individual cases.

## 5 Conclusion

In summary, thrombotic microangiopathy (TMA) constitutes a severe adverse drug reaction that may lead to fatal outcomes. Despite its life-threatening nature, the associated risks remain insufficiently characterized. This study conducted a comprehensive evaluation of TMA-associated medications using the FDA Adverse Event Reporting System (FAERS) database, followed by systematic analysis of the top 30 drugs exhibiting the strongest safety signals. For clinical practice, we recommend enhanced pharmacovigilance measures to mitigate TMA risk during drug administration.

## Data Availability

The original contributions presented in the study are included in the article/Supplementary Material, further inquiries can be directed to the corresponding author.
